# Pulmonary alveolar proteinosis after lung transplantation

**DOI:** 10.1002/rcr2.566

**Published:** 2020-05-05

**Authors:** Chandima Divithotawela, Simon H. Apte, Maxine E. Tan, Tharushi A. De Silva, Daniel C. Chambers

**Affiliations:** ^1^ QLD Lung Transplant Service The Prince Charles Hospital Brisbane QLD Australia; ^2^ School of Clinical Medicine The University of Queensland Brisbane QLD Australia; ^3^ School of Biomedical Sciences Queensland University of Technology Brisbane QLD Australia

**Keywords:** Interleukin‐1β, lipofuscin, lung transplant, pulmonary alveolar proteinosis, whole lung lavage

## Abstract

We report the case of a 69‐year‐old man five‐month post double lung transplant for idiopathic pulmonary fibrosis (IPF) who presented with progressive breathlessness, loss of lung function, and diffuse ground glass shadowing on the chest computed tomography. Transbronchial lung biopsy revealed foamy macrophages, hyperplasia of type II pneumocytes, and eosinophilic material in the alveolar space. Video thoracic lung biopsy was performed, and histology confirmed pulmonary alveolar proteinosis. Anti‐granulocyte‐macrophage colony‐stimulating factor (GM‐CSF) antibodies were negative. Bilateral sequential whole lung lavage (WLL) was performed. Lavage fluid recovered during WLL was notably dark brown in colour and upon analysis was shown to contain heavily oxidized protein (lipofuscin), giant lipofuscin‐engorged macrophages, and a highly pro‐inflammatory gene expression profile. Following WLL, the patient's symptoms, lung function, and radiology appearance improved. His repeat bronchoalveolar lavage (BAL) fluid analysis showed reduced lipofuscin and normalized macrophage size and gene expression.

## Graphical abstract

Here, we report a case of secondary pulmonary alveolar proteinosis (PAP) after double lung transplantation with highly oxidized protein and pro‐inflammatory gene expression in whole lung lavage (WLL) fluid. Our patient was successfully treated with WLL. His repeat bronchoalveolar lavage (BAL) fluid analysis showed reduced lipofuscin and normalized macrophage size and gene expression.

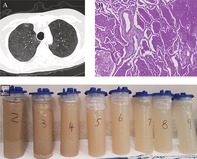

## Introduction

Pulmonary alveolar proteinosis (PAP) occurs due to the accumulation of surfactant‐like lipoproteinaceous material in the alveolar space due to macrophage dysfunction [1]. There are three main forms: congenital (2%), secondary (5–10%), and primary (90%) [1]. The congenital form occurs due to mutations in the genes coding for surfactant protein or the granulocyte‐macrophage colony‐stimulating factors (GM‐CSF) receptor [2]. Underlying conditions that can cause impairment of macrophage function such as immunosuppression, haematological cancers, and silica dust can produce the secondary form [1]. The most prevalent form, primary PAP, is due to an antibody against GM‐CSF [2].

PAP after lung transplantation is very rare. Out of 39,835 lung transplants between 1985 and 2011, only eight cases of PAP have been reported in the literature [3]. Here, we report a case of secondary PAP after double lung transplantation with highly oxidized protein and pro‐inflammatory gene expression in whole lung lavage (WLL) fluid. The patient was treated successfully with WLL.

## Case Report

A 69‐year‐old man underwent bilateral sequential lung transplantation for idiopathic pulmonary fibrosis (IPF). The immediate post‐transplant period was uncomplicated. He was maintained on a routine tacrolimus, prednisolone, and mycophenolate mofetil immunosuppression regime.

**Figure 1:**
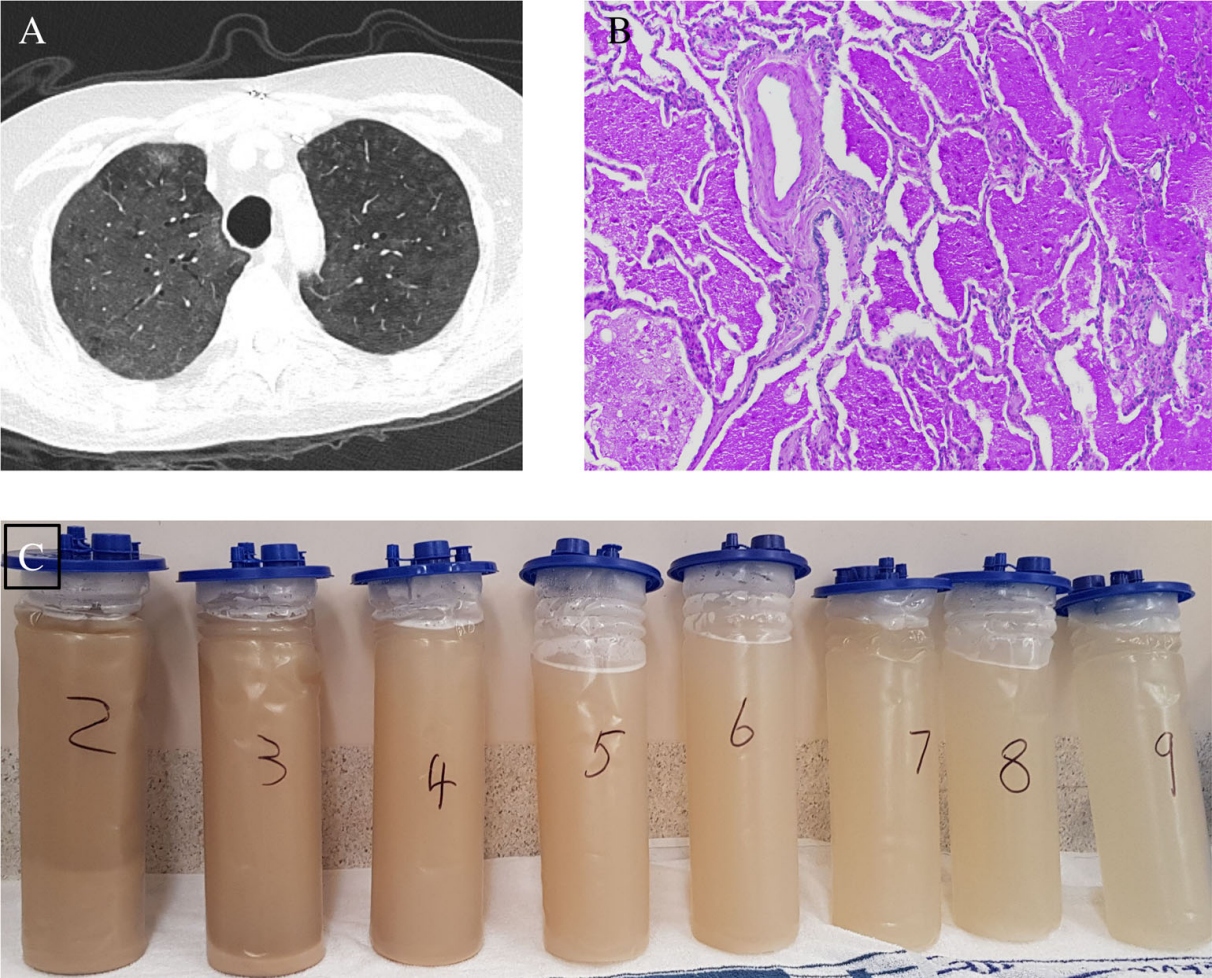
(A) Computed tomography (CT) scan of the patient three months post‐transplant showing ground‐glass opacities and interlobular septal thickening giving a “crazy‐paving” appearance characteristic of pulmonary alveolar proteinosis. (B) Open lung biopsy confirms alveolar proteinosis with accumulation of periodic acid‐Schiff (PAS) staining positive and diastase‐resistant intra‐alveolar material. (C) Sequential alveolar returns following whole lung lavage, demonstrating gradual clearing (each container is 3 L).

In the first six months, he developed recurrent pulmonary infections with *Pseudomonas aeruginosa*, *Enterobacter cloacae*, *Aspergillus fumigatus*, KI human polyomavirus, adenovirus, and *Mycobacterium intracellulare*. At three months post‐transplant, his course was further complicated with an acute rejection episode and the appearance of diffuse ground‐glass opacities (GGOs) on his computed tomography (CT) chest (Fig. 1A). Hypogammaglobulinaemia was corrected. Despite treatment of the infectious complication and the episode of rejection, he continued to deteriorate with worsening breathlessness and deteriorating lung function and the diffuse pulmonary GGO persisted. Repeat transbronchial biopsy was performed which showed patchy intra‐alveolar eosinophilic granular secretions. Subsequently, a video‐assisted thoracotomy right lower lobe lung biopsy showed patchy but widespread intra‐alveolar accumulation of periodic acid‐Schiff (PAS)‐positive material consistent with alveolar proteinosis (Fig. 1B). Anti GM‐CSF antibodies were negative.

**Figure 2:**
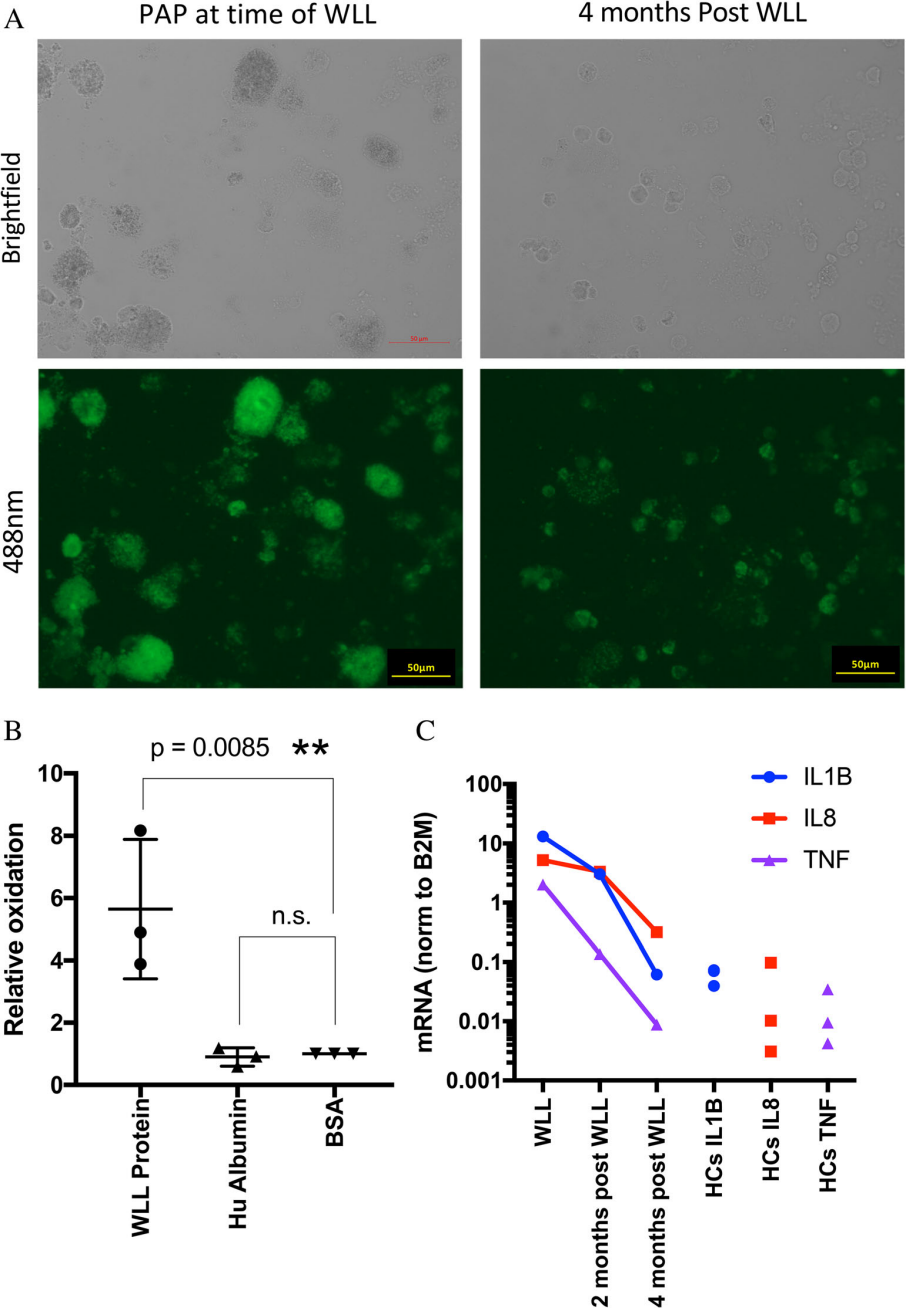
(A) Brightfield and 488 nm excited images of cells recovered from lung fluid prior to and four months after whole lung lavage (WLL). Enlarged alveolar macrophages are engorged with oxidized protein (green fluorescence). Macrophages are smaller in size and are less intensely fluorescent four months after WLL. (B) Oxidation level of the proteinaceous precipitate from WLL was assessed using Oxyblot (Merck Millipore, Australia) and compared to bovine serum albumin (BSA) and human albumin controls (three repeat experiments shown, one‐way analysis of variance (ANOVA); Prism, Australia). (C) mRNA expression of the inflammatory cytokines interleukin (IL)‐1β, IL‐8, and tumour necrosis factor alpha (TNFα) (quantitative polymerase chain reaction, qPCR; Taqman, Australia) in the cellular fraction recovered from lavage fluid at the time of WLL and two and four months later. Expression levels from three healthy volunteers are also shown. ** *P* < 0.05 significant.

WLL was performed in a staged manner, with the contralateral lung lavaged 11 days later. As the PAP was felt likely secondary to immunosuppression‐induced macrophage dysfunction, the immunosuppression regime was adjusted to the lowest acceptable level. The WLL fluid was notably dark brown in colour with dense proteinaceous precipitate (Fig. 1C). Examination under fluorescent microscope revealed masses of highly autofluorescent protein aggregates and giant fluorescent protein‐engorged macrophages (Fig. 2A). Under normal brightfield examination, these macrophages appeared to be full of dark‐brown material that could be mistaken for haemosiderin; however, the samples were Perls' stain negative. Oxyblot analysis (Fig. 2B) confirmed the material consisted of oxidized aggregates of highly fluorescent protein known as lipofuscin. At the time of the WLL, a highly pro‐inflammatory gene expression profile (interleukin (IL)‐1β, IL‐8, and tumour necrosis factor (TNF)) was evident (Fig. 2C); however, after WLL, inflammatory cytokine levels returned to those of healthy controls and pro‐inflammatory gene expression was reduced. After WLL, transbronchial biopsy did not show any intra‐alveolar eosinophilic material and the patient's symptoms, lung function, and radiological appearance improved back to his baseline level.

## Discussion

Here, we report a rare occurrence of secondary PAP in a lung transplant patient which was successfully treated with WLL.

Patients with PAP commonly present with exertional dyspnoea and cough [2]. The most common examination finding is inspiratory crackles. CT chest will show patchy ground‐glass changes with interlobular septal thickening giving rise to a crazy‐paving pattern [2]. After lung transplantation, there are many common causes of pulmonary GGO, including infection and rejection making the PAP diagnosis challenging [4]. In the early course of his disease, most of his clinical and radiological manifestations were attributed to rejection and infection episodes. Perhaps, a further clue was the presence of intra‐alveolar material on transbronchial biopsy which was initially overlooked. An open lung biopsy is the gold standard for PAP diagnosis [2], and was definitive in our patient.

Although post‐transplant infectious complications are common, the number and diversity of infectious complications experienced by our patient perhaps points to a defect of pulmonary innate immune function and PAP. PAP increases the risk of bacterial, viral, mycobacterial, and fungal pulmonary infections [2, 4, 5]. This is thought to be secondary to impaired macrophage chemotaxis, adhesion, phagocytosis, and microbicidal activity [2]. This could explain the recurrent infections that were seen in our patient.

In the primary and congenital forms of PAP, GM‐CSF stimulation of alveolar macrophages is defective due to autoantibodies to GM‐CSF or genetic defects in GM‐CSF, respectively. Our patient was negative for autoantibodies to GM‐CSF and congenital defects were considered unlikely so gene sequencing was not performed [2]. In the secondary form of PAP that occurs in the post‐transplant setting, the aetiology is attributed to an immunosuppression‐related defect in alveolar macrophage number and function [3]. Hence, reduction of immunosuppression is considered as an initial step in treatment. Successful treatment of two patients with post lung transplant secondary PAP after reducing baseline immunosuppression has been reported [3]; however; in four other patients this was not successful [4]. As in our patient, in the presence of recurrent acute rejections, minimizing immunosuppression treatment could be challenging [4].

Excessive amounts of surfactant accumulate in the alveolar space in PAP due to reduced macrophage clearance [2]. In our patient, analysis of alveolar fluid confirmed that the excess proteinaceous material was highly oxidized and had formed aggregates of lipofuscin. The macrophages from our patient were heavily engorged with lipofuscin but were still able to phagocytose latex beads (data not shown). Lipofuscin is resistant to catabolism by the proteasome and inhibits the ability of the proteasome to catabolize other (unoxidized) proteins further increasing protein accumulation [6]. Furthermore, a component of lipofuscin (N‐retinylidene‐N‐retinylethanolamine) has been shown to trigger activation of the inflammasome complex and induce production of the potently pro‐inflammatory cytokine IL‐1β [7].

We hypothesize that an initial oxidative insult in our patient, for example, ischaemia–reperfusion at the time of transplantation, or an infectious event, may have led to initial oxidative stress, accumulation of lipofuscin, and subsequent triggering of inflammasome activation and further inflammation and oxidative stress. This initial accumulation may then have perpetuated a vicious cycle of lipofuscin accumulation. Bulk removal of the accumulated indigestible oxidized protein by WLL was required to break the cycle.

WLL is the mainstay of treatment in persistent, progressive PAP [2]. WLL improves clinical, physiological, and radiological manifestations, but has mainly been used in primary PAP with only case reports in secondary PAP [4]. Here, we have demonstrated the successful use of WLL to treat PAP after lung transplantation.

### Disclosure Statement

Appropriate written informed consent was obtained for publication of this case report and accompanying images.
